# Long-Term Changes in Salivary Oxidative and Nitrosative Stress Biomarkers After Photodynamic Therapy Versus Clobetasol in Oral Lichen Planus: A Randomized Clinical Trial

**DOI:** 10.3390/antiox15101259

**Published:** 2026-10-01

**Authors:** Magdalena Sulewska, Patryk Wiśniewski, Jagoda Tomaszuk, Aleksandra Pietruska, Anna Zalewska, Emilia Szymańska, Katarzyna Winnicka, Mateusz Maciejczyk, Małgorzata Żendzian-Piotrowska, Małgorzata Pietruska

**Affiliations:** 1 Department of Periodontal and Oral Mucosa Diseases, Medical University of Bialystok, 15-269 Bialystok, Poland; jagoda.tomaszuk@umb.edu.pl (J.T.); malgorzata.pietruska@umb.edu.pl (M.P.); 2 Private Dental Practice, 1/34 Waszyngtona Street, 15-269 Białystok, Poland; perio@umb.edu.pl; 3 Department of Restorative Dentistry, Medical University of Bialystok, 15-089 Bialystok, Poland; anna.zalewska1@umb.edu.pl; 4 Independent Laboratory of Experimental Dentistry, Medical University of Bialystok, 15-089 Bialystok, Poland; 5 Department of Pharmaceutical Technology, Medical University of Białystok, Mickiewicza 2c, 15-222 Białystok, Poland; emilia.szymanska@umb.edu.pl (E.S.); katarzyna.winnicka@umb.edu.pl (K.W.); 6 Department of Hygiene, Epidemiology and Environmental Health, Medical University of Bialystok, Mickiewicza 2c, 15-222 Białystok, Poland; mat.maciejczyk@gmail.com (M.M.); mzpiotrowska@gmail.com (M.Ż.-P.)

**Keywords:** oral lichen planus, photodynamic therapy, corticosteroid therapy, oxidative stress, nitrosative stress, saliva

## Abstract

**Background:** Oxidative and nitrosative stress may contribute to the pathogenesis of oral lichen planus (OLP). This study compared the effects of photodynamic therapy (PDT) and topical corticosteroid therapy (CT) on selected salivary redox biomarkers. **Materials and Methods:** Thirty-nine patients with OLP were randomized to five weekly sessions of 5-aminolevulinic acid PDT (*n* = 20) or topical clobetasol applied for 14 days (*n* = 19). Salivary advanced oxidation protein products (AOPP), advanced glycation end products (AGE), 3-nitrotyrosine (NT), and peroxynitrite (PN) were measured before treatment, immediately after treatment, and after 6 and 12 months. Associations with lesion area and pain intensity were also assessed. **Results:** PDT significantly reduced AOPP concentrations, which remained below baseline throughout follow-up and were lower than after CT at all post-treatment assessments. AGE increased transiently after PDT but decreased thereafter and was lower than after CT at 12 months. NT was lower after PDT than after CT immediately after treatment. PN concentrations showed significant within-group changes over time in the CT group; however, baseline PN concentrations differed between the treatment groups, limiting direct interpretation of between-group differences. Biomarker concentrations showed no consistent associations with lesion area or pain intensity. **Conclusions:** PDT and CT were associated with different patterns of change in selected salivary redox biomarkers in OLP. PDT was particularly associated with a sustained reduction in AOPP concentrations, whereas the investigated biomarkers showed no consistent relationship with clinical disease severity.

## 1. Introduction

Oral lichen planus (OLP) is a chronic, immune-mediated inflammatory disease of the oral mucosa classified among oral potentially malignant disorders. It affects approximately 1% of the general population, predominantly middle-aged and older women [1,2]. The clinical presentation ranges from asymptomatic reticular lesions to erythematous and erosive forms associated with pain, burning sensations, and impaired quality of life [3].

The etiopathogenesis of OLP has not yet been fully elucidated. A central role is attributed to a T-cell-mediated immune response, particularly the activation of cytotoxic CD8+ T lymphocytes, which induce apoptosis of basal keratinocytes and contribute to the persistence of chronic inflammation [4,5,6]. Increasing evidence also indicates that oxidative stress is involved in the development and progression of OLP. Excessive production of reactive oxygen species (ROS) by activated inflammatory cells, accompanied by insufficient antioxidant defense, may result in oxidative damage to proteins, lipids, and nucleic acids, further enhancing keratinocyte apoptosis and inflammatory signaling [7,8].

Nitrosative stress may represent another important component of OLP pathogenesis. Previous studies, including a systematic review and meta-analysis, have demonstrated increased nitric oxide (NO) levels in the saliva and blood of patients with lichen planus [9,10]. In an oxidative environment, NO rapidly reacts with the superoxide anion to form peroxynitrite (ONOO^−^), a highly reactive oxidizing and nitrating agent. Therefore, the simultaneous overproduction of NO and superoxide in OLP may favor increased peroxynitrite formation and the development of nitrative stress. Peroxynitrite (PN) can oxidize cellular components and nitrate protein tyrosine residues, resulting in the formation of 3-nitrotyrosine (NT), which is widely used as a marker of protein nitration [11,12].

Oxidative and carbonyl damage may also be reflected by advanced oxidation protein products (AOPPs) and advanced glycation end products (AGEs). AOPPs are a heterogeneous group of relatively stable products formed during oxidative modification and cross-linking of proteins; in this context, “advanced” refers to later-stage, accumulated oxidation products rather than disease severity. Increased salivary AOPP levels have been reported in patients with OLP [13]. AGEs are likewise heterogeneous, relatively stable end products formed during the later stages of non-enzymatic glycation and glycoxidation of proteins, lipids, and nucleic acids [14]. Both AOPPs and AGEs may not only reflect accumulated molecular damage but also amplify oxidative stress and inflammatory responses, thereby potentially contributing to the maintenance of chronic oral mucosal inflammation [15,16].

Topical corticosteroid therapy (CT) remains the gold standard for the symptomatic treatment of OLP and is effective in reducing lesion severity and pain intensity [17,18]. However, prolonged or repeated corticosteroid use may be associated with adverse effects, including oral candidiasis, mucosal atrophy, delayed healing, and impaired salivary secretion [19]. Moreover, disease recurrence after treatment discontinuation is common, emphasizing the need for safe and effective alternative therapeutic approaches.

In recent years, photodynamic therapy (PDT) has emerged as a promising, minimally invasive treatment option for OLP. PDT involves the topical application of a photosensitizer followed by irradiation with light at a wavelength selected according to the absorption characteristics of the photosensitizer. In ALA-mediated PDT, 5-aminolevulinic acid is metabolized intracellularly to protoporphyrin IX (PpIX), which can be effectively excited with red light at approximately 630 nm. In the presence of molecular oxygen, this reaction leads to the local generation of reactive species and induces cytotoxic and immunomodulatory effects within the treated tissue [20]. Importantly, this transient and localized generation of reactive species represents an integral part of the photochemical mechanism of PDT and differs from the sustained oxidative and nitrative imbalance associated with chronic inflammation in OLP. Thus, assessment of redox biomarkers after treatment may reflect the subsequent net effect of PDT on the oral redox environment rather than the immediate generation of reactive species during irradiation. Clinical studies have shown that PDT can significantly reduce lesion size and pain intensity, with outcomes comparable to those achieved with topical corticosteroids and a generally favorable safety profile [21,22]. Moreover, long-term observations have demonstrated sustained reductions in lesion extent, clinical severity, and pain following PDT, suggesting that its therapeutic effects may persist beyond the immediate treatment period [23,24].

Recent studies have indicated that both PDT and corticosteroid therapy may influence the salivary redox profile in patients with OLP. Treatment-related changes have been observed in global oxidative stress indices, including total oxidant status, total antioxidant capacity, and oxidative stress index, as well as in selected components of the salivary antioxidant system [25,26]. These findings suggest that the clinical effects of both therapies may be accompanied by modulation of the local redox environment. However, previous investigations have focused primarily on global redox parameters and antioxidant defense mechanisms rather than on products reflecting oxidative, glycoxidative, and nitrative molecular damage.

Saliva is a useful biological material for evaluating these processes because its collection is non-invasive, inexpensive, and easily repeatable. As saliva remains in direct contact with oral mucosal lesions, its composition may reflect biochemical alterations occurring locally within the affected tissues and may be useful for monitoring treatment-related changes [27,28].

Although increased salivary levels of NO and AOPPs have previously been reported in patients with OLP, there are currently no data on the effects of PDT and corticosteroid therapy on salivary markers of nitrative stress or on AOPP and AGE concentrations. In particular, it remains unknown whether these therapies influence peroxynitrite formation, protein nitration, oxidative protein damage, and glycoxidative processes, and whether changes in these parameters are associated with clinical improvement.

The present study extends our previous works using the same therapeutic protocols, which separately evaluated the clinical effectiveness of 5-aminolevulinic acid-mediated photodynamic therapy versus topical corticosteroid therapy and changes in other salivary markers of oxidative stress. In contrast, the current analysis focuses specifically on markers of oxidative protein damage, glycoxidation, and nitrative stress, thereby addressing a distinct aspect of the redox alterations associated with these treatments.

Therefore, the primary aim of the present randomized clinical study was to evaluate and compare the effects of photodynamic therapy and topical corticosteroid therapy on salivary concentrations of AOPPs, AGEs, 3-nitrotyrosine, and peroxynitrite in patients with OLP. The secondary aim was to assess the relationships between changes in the concentrations of these biomarkers and changes in lesion size and pain intensity measured using the visual analog scale.

## 2. Materials and Methods

### 2.1. Study Participants

The present study was conducted as part of a previously reported single-center, prospective, randomized clinical trial comparing photodynamic therapy with topical corticosteroid therapy in patients with OLP [22]. The clinical outcomes of the trial, including lesion size and pain intensity, have been reported previously and are used in the present study only for correlation analyses with salivary biomarkers [22]. Importantly, the salivary concentrations of AOPPs, AGEs, 3-nitrotyrosine, and peroxynitrite analyzed in the present study have not been reported previously and represent a new dataset. Although the study cohort partially overlaps with those included in our previous publications evaluating other salivary markers of oxidative stress [25,26], none of the biomarkers investigated in the present analysis were included in those reports. Thus, the present study addresses a distinct aspect of redox biology, focusing specifically on oxidative protein damage, glycoxidation, and nitrative stress.

The trial was carried out at the Department of Periodontal and Oral Mucosa Diseases, Medical University of Bialystok in compliance with the EU Directive 2001/20/EC and the ICH GCP. The study followed the ethical principles outlined in the Declaration of Helsinki, and the study protocol was approved by the Bioethics Committee of the Medical University of Bialystok (approval no. APK.002.372.2021). The trial was retrospectively registered at ClinicalTrials.gov (NCT07536737; 15 April 2026), as prospective trial registration was not required at the time the study was initiated.

Before enrollment, all participants received detailed information regarding the study procedures and provided written informed consent. The trial was conducted and reported in accordance with the CONSORT 2010 guidelines [29].

The present analysis included 39 patients with clinically and histopathologically confirmed OLP, comprising 32 women and 7 men. The mean age of the participants was 59.64 ± 9.44 years. Eligible patients were adults aged ≥18 years with a diagnosis of OLP confirmed by histopathological examination. Exclusion criteria comprised pregnancy or breastfeeding, severe systemic conditions, including malignant, dermatological, and hepatic diseases, photosensitivity or known hypersensitivity to the photosensitizer, OLP treatment during the preceding 6 months, use of immunosuppressive or immunomodulatory medications, psychiatric disorders, active periodontitis, and the presence of other oral mucosal diseases. Diabetes mellitus was not considered an exclusion criterion provided that it was well controlled, as determined on the basis of the patient’s medical history- however, none of the enrolled patients had diabetes mellitus. None of the participants were smokers. In addition, no abnormalities in salivary flow rate were observed in any participant at the time of saliva collection.

### 2.2. Study Groups

Participants were allocated to either the PDT group or the CT group using simple randomization. The allocation sequence was prepared in Microsoft Excel for Microsoft 365 and was managed by an independent investigator who was not involved in the clinical assessment.

**Figure 1:**
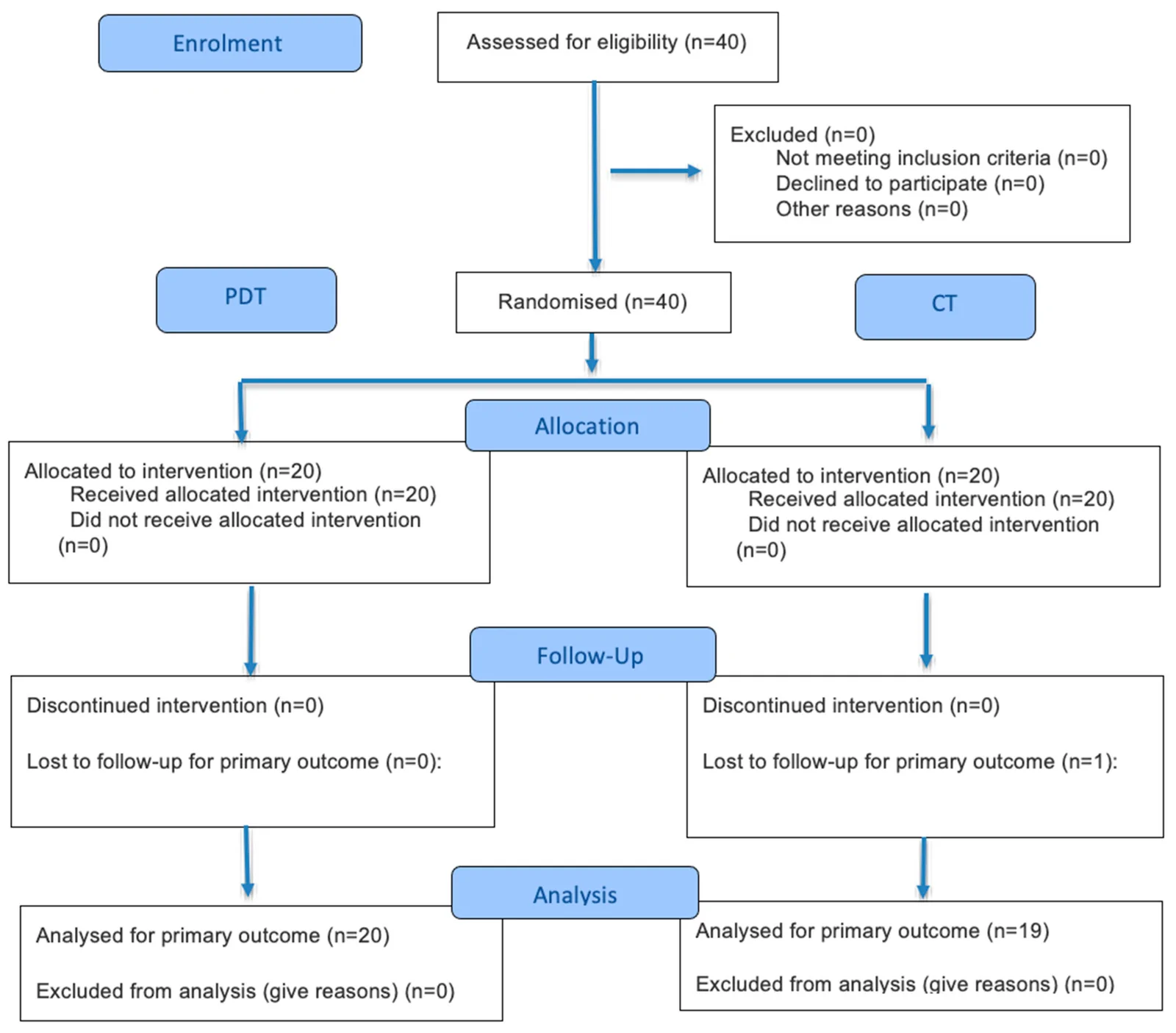
CONSORT flow diagram showing participant enrolment, randomization, allocation to photodynamic therapy (PDT) or topical corticosteroid therapy (CT), follow-up, and inclusion in the final analysis.

A single-blind study design was applied. The examiner responsible for evaluating the clinical outcomes was unaware of treatment allocation and had no access to information regarding the intervention received by each participant. The examiner was instructed not to discuss treatment details with the patients, who were likewise asked not to disclose their assigned therapy. Blinding of the participants was not feasible because of the distinct nature and administration of the two interventions (Figure 1).

Patients assigned to the PDT group were treated with a mucoadhesive emulgel containing 5% (*w*/*w*) 5-aminolevulinic acid (patent EP4665304A1), according to the previously described ALA-PDT protocol [22]. After the oral mucosa had been dried, the preparation was applied to the lesion and surrounding mucosa as an approximately 2 mm thick layer at 40 and 20 min before irradiation. The treated area was covered with gauze and sterile compresses to reduce contact with saliva.

Irradiation was performed using a FotoSan^®^ 630 LED device (CMS Dental A/S, Roslev, Denmark) at a wavelength of 630 nm, a power output of 300 mW, and an energy density of 108 J/cm^2^. The light was delivered continuously in a non-contact mode, with the applicator positioned approximately 2 mm from the lesion, for 6 min per cm^2^ of the treated area. The PDT protocol consisted of five sessions administered at weekly intervals.

Patients allocated to the CT group received topical clobetasol propionate at a concentration of 0.5 mg/g (Clobederm), applied twice daily for 14 consecutive days.

### 2.3. Clinical Assessment

Clinical evaluations were performed at four time points: before treatment initiation (T0), immediately after completion of the assigned treatment protocol (T1), and at 6-month (T6) and 12-month (T12) follow-up visits. Because the treatment protocols differed in duration, T1 corresponded to 5 weeks after treatment initiation in the PDT group and 2 weeks after treatment initiation in the CT group. Thus, T1 represented the immediate post-treatment assessment in both groups but was not a time-matched assessment. At each assessment, the oral lesions were examined macroscopically and documented using standardized clinical photographs.

Lesion dimensions were measured with a periodontal probe (PCPUNC 15; Hu-Friedy). For each lesion, the maximum length and width were recorded as the longest distances between its most peripheral margins and the adjacent clinically healthy mucosa. These measurements were subsequently used to calculate lesion area, expressed in cm^2^.

All clinical measurements were performed by the same examiner, who remained blinded to treatment allocation. Before the study, the examiner was calibrated using a separate group of 10 patients who were not included in the trial. Measurements were repeated after 24 h, with an acceptable difference between paired measurements defined as ≤0.5 cm.

At each visit, participants also completed a questionnaire concerning subjective symptoms associated with the oral lesions. The intensity of pain, burning, and itching was assessed using a visual analog scale (VAS). For descriptive analysis, VAS scores were classified as follows: 0, no symptoms; 1–3, mild symptoms; 4–6, moderate symptoms; 7–9, severe symptoms; and 10, the worst imaginable pain [30].

### 2.4. Saliva Collection

Unstimulated whole saliva was obtained at four predefined study visits: prior to treatment (T0), after completion of the assigned intervention (T1), and at 6 months (T6), and 12 months (T12) after treatment. Owing to the different duration of the therapeutic protocols, the T1 assessment was performed 5 weeks after the first PDT session and 2 weeks after the initiation of corticosteroid therapy.

To minimize the influence of external factors on salivary composition, all participants followed standardized preparation instructions. They were asked not to consume food or beverages other than water for at least 2 h before the visit, to refrain from oral hygiene procedures before sampling, and to avoid medication intake for a minimum of 8 h. Collection was scheduled between 8:00 and 10:00 a.m. and was conducted under the same environmental conditions in a separate examination room.

Before sampling, each participant remained seated for 5 min with the head slightly bent forward. The mouth was then rinsed with distilled water. Saliva secreted during the first minute was not retained. Thereafter, participants expectorated unstimulated saliva into sterile Falcon tubes for a maximum of 15 min, until approximately 5 mL had been collected.

The specimens were immediately transferred onto ice and centrifuged at 3000× *g* for 20 min at 4 °C. Following centrifugation, the supernatant was separated and stored at −80 °C until biochemical measurements were performed.

### 2.5. Biomarkers Assay

AOPP were measured spectrophotometrically using the method described by Kalousová et al. [31]. Saliva samples were diluted fivefold with 0.02 M phosphate-buffered saline (PBS), and the oxidation of iodide ions was evaluated by recording absorbance at 340 nm. AOPP concentrations were normalized to the total protein content and expressed as pmol/mg protein.

AGEs were quantified according to the spectrofluorometric procedure developed by Kalousová et al. [31]. The saliva samples were diluted with 0.02 M PBS at a ratio of 1:5 (*v*/*v*). Fluorescence intensity was measured at an excitation wavelength of 350 nm and an emission wavelength of 440 nm. AGE levels were reported as arbitrary fluorescence units per milligram of total protein (AFU/mg protein). This spectrofluorometric assay reflects the overall fluorescence signal of fluorescent AGE-related compounds rather than the concentration of a specific AGE molecule and may also be influenced by other fluorescent constituents present in the sample. Accordingly, the obtained values should be interpreted as an aggregate measure of AGE-associated fluorescence rather than a specific molecular AGE determination.

Salivary PN concentrations were determined using a colorimetric method based on peroxynitrite-dependent nitration and the formation of nitrophenol [32]. Absorbance was measured at 320 nm, and the obtained values were normalized to the total protein content and expressed as nmol/mg protein.

Salivary NT concentrations were quantified using a colorimetric enzyme-linked immunosorbent assay and a commercially available diagnostic kit (Immundiagnostik AG, Bensheim, Germany). The procedure was performed in accordance with the manufacturer’s instructions. The results were normalized to the total protein content and expressed as nmol/mg protein.

Total protein concentration was determined using the bicinchoninic acid method with the Pierce BCA Protein Assay Kit (Thermo Scientific, Rockford, IL, USA).

### 2.6. Statistical Analysis

An a priori sample size calculation was performed using G*Power version 3.1 (Heinrich Heine University Düsseldorf, Düsseldorf, Germany). The calculation was based on an F test for repeated-measures analysis of variance with a within–between interaction. Assuming an effect size of *f* = 0.20, α = 0.05, and a statistical power of 0.80, the minimum required sample size was estimated at 36 participants. To account for an anticipated dropout rate of approximately 10%, the planned sample size was increased to 40 patients.

Statistical analyses were conducted using GraphPad Prism version 11.0.0 (GraphPad Software, Boston, MA, USA). Data distribution was examined using the Shapiro–Wilk test. As the analyzed variables did not follow a normal distribution, nonparametric statistical methods were applied. Results were reported as medians because of the non-normal distribution and the wide ranges between minimum and maximum values, which could substantially influence arithmetic means.

Changes in biomarker concentrations and clinical parameters over time within each treatment group were analyzed using the Friedman test, followed by Dunn’s multiple-comparison test with Bonferroni correction. Differences between the PDT and CT groups at individual study time points were assessed using the Mann–Whitney U test.

Associations between salivary biomarker concentrations and clinical outcomes, including lesion area and pain intensity assessed using the visual analog scale, were evaluated using Spearman’s rank correlation coefficient. No post hoc power calculation was performed specifically for these correlation analyses; therefore, the resulting associations were considered exploratory and should be interpreted accordingly.

Statistical significance was defined as a two-sided *p* value < 0.05.

## 3. Results

Initially, 40 patients were randomized equally between the two treatment groups, with 20 participants assigned to each group. One patient from the CT group was lost to follow-up; therefore, the final analysis included 39 patients with OLP, comprising 32 women and 7 men (Table 1). The final group sizes were 20 patients in the PDT group and 19 patients in the CT group. Salivary concentrations of AOPP, AGE, NT, and PN were assessed at baseline (PDT0 and CT0), immediately after completion of treatment (PDT1 and CT1), and at the 6-month (PDT6 and CT6) and 12-month (PDT12 and CT12) follow-up visits.

### 3.1. Time-Dependent Changes in Salivary Biomarkers in the PDT Group

**Figure 2:**
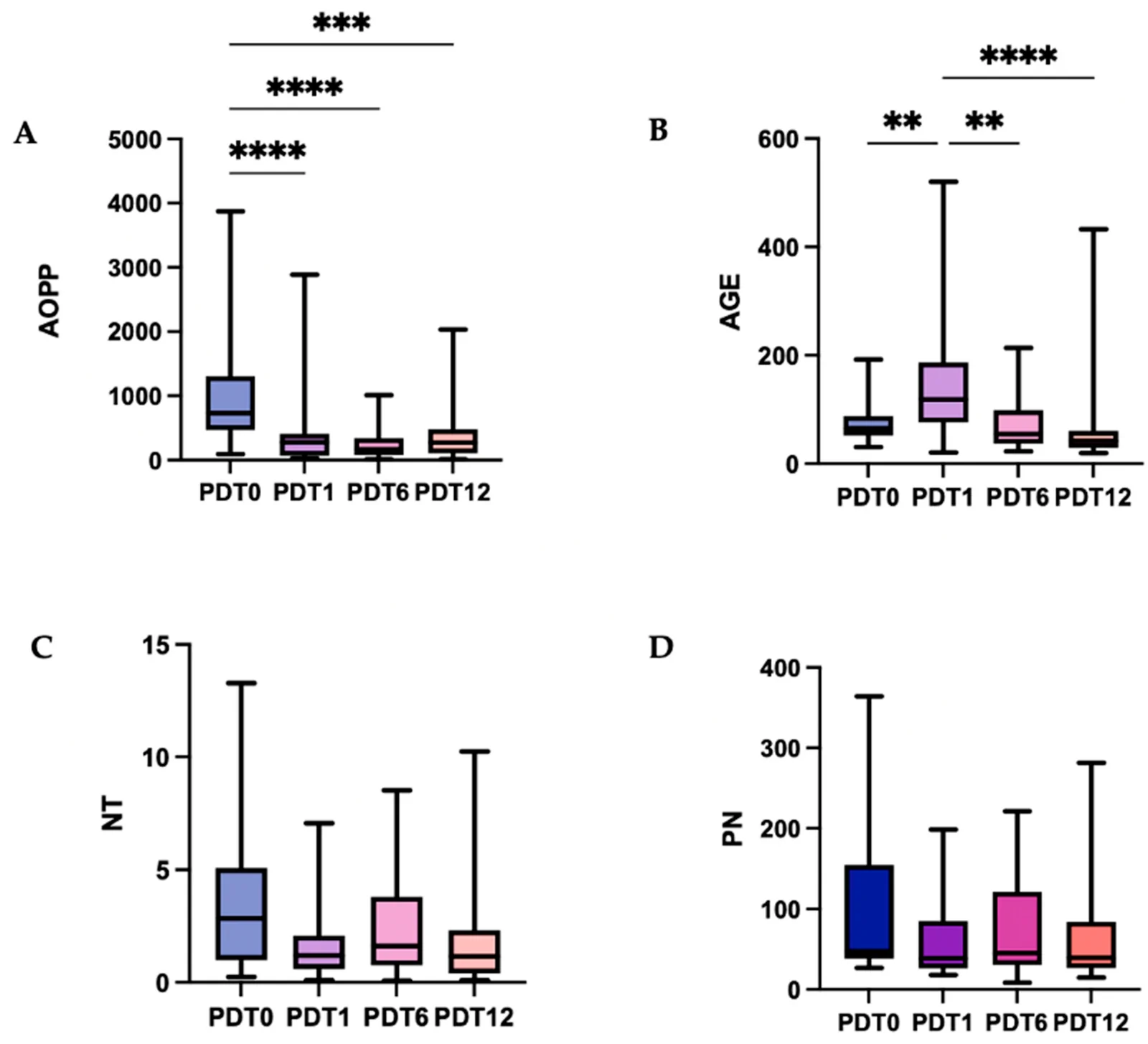
Time-dependent changes in salivary biomarker concentrations in the PDT group. Box-and-whisker plots show concentrations of (**A**) AOPP, (**B**) AGE, (**C**) NT, and (**D**) PN at baseline (PDT0), immediately after treatment (PDT1), and at 6-month (PDT6) and 12-month (PDT12) follow-ups. Statistical significance was assessed using the Friedman test followed by Dunn’s multiple-comparison test with Bonferroni correction. Y-axis scaling differs between panels for clarity. ** *p* < 0.01, *** *p* < 0.001, **** *p* < 0.0001.

In the PDT group, AOPP concentrations changed significantly over the observation period (*p* < 0.0001). The median AOPP level decreased from 732.9 pmol/mg at T0 to 277.1 pmol/mg at T1 and reached its lowest value at T6 (167.7 pmol/mg). At T12, the median increased slightly to 275.0 pmol/mg but remained significantly lower than the baseline value. Post hoc analysis demonstrated significant differences between T0 and T1 (*p* < 0.0001), T0 and T6 (*p* < 0.0001), and T0 and T12 (*p* < 0.001) (Table 2, Figure 2).

A significant time-dependent variation was also observed for AGE (*p* < 0.0001). The median AGE level increased from 65.38 AFU/mg at T0 to 118.6 AFU/mg immediately after treatment, representing a significant increase (*p* < 0.01). Subsequently, AGE decreased to 54.55 AFU/mg at T6 and 42.15 AFU/mg at T12. Compared with T1, the reductions observed at T6 and T12 were statistically significant (*p* < 0.01 and *p* < 0.0001, respectively).

NT concentrations differed significantly across the study visits in the overall Friedman analysis (*p* = 0.0464). The median decreased from 2.843 nmol/mg at T0 to 1.191 nmol/mg at T1, followed by values of 1.613 nmol/mg at T6 and 1.150 nmol/mg at T12. However, none of the pairwise comparisons remained statistically significant after correction for multiple testing.

No significant changes in PN concentrations were detected over time (*p* = 0.1032). The median PN concentration was 47.57 nmol/mg at T0, 38.74 nmol/mg at T1, 45.27 nmol/mg at T6, and 39.63 nmol/mg at T12.

### 3.2. Time-Dependent Changes in Salivary Biomarkers in the CT Group

In the CT group, no significant changes in AOPP concentrations were observed over the study period (*p* = 0.4218). The median AOPP concentration increased from 828.9 pmol/mg at T0 to 936.1 pmol/mg at T1, followed by a decrease to 547.6 pmol/mg at T6 and 478.3 pmol/mg at T12.

AGE concentrations also remained statistically unchanged (*p* = 0.3633). Median values were 68.58 AFU/mg at T0, 59.89 AFU/mg at T1, 56.54 AFU/mg at T6, and 69.20 AFU/mg at T12. Similarly, no significant time-dependent differences were found for NT (*p* = 0.4538), although the median concentration decreased from 2.107 nmol/mg at baseline to 1.171 nmol/mg at T12.

**Figure 3:**
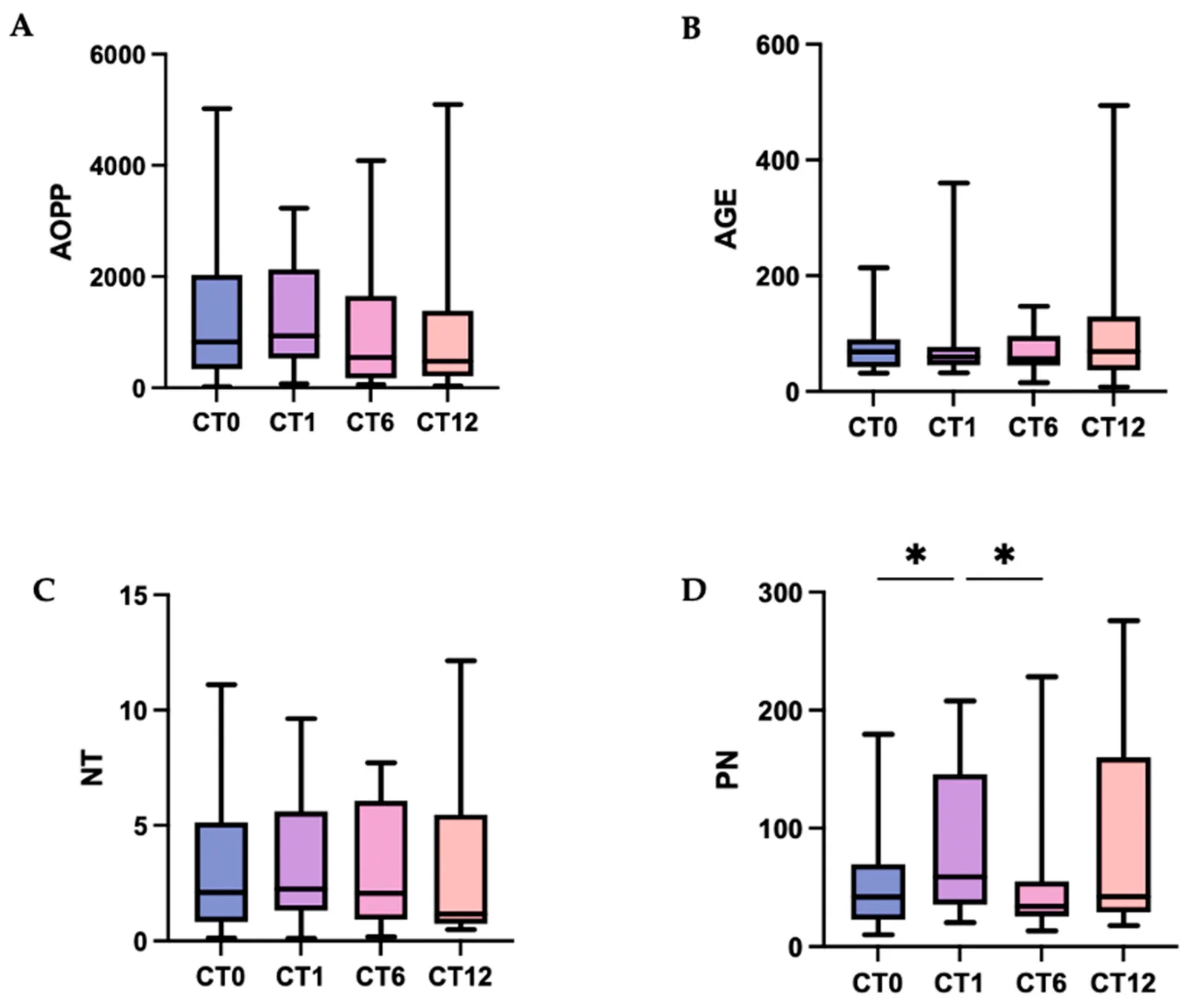
Time-dependent changes in salivary biomarker concentrations in the CT group. Box-and-whisker plots show concentrations of (**A**) AOPP, (**B**) AGE, (**C**) NT, and (**D**) PN at baseline (CT0), immediately after treatment (CT1), and at 6-month (CT6) and 12-month (CT12) follow-ups. Statistical significance was assessed using the Friedman test followed by Dunn’s multiple-comparison test with Bonferroni correction. Y-axis scaling differs between panels for clarity. * *p* < 0.05.

In contrast, PN concentrations varied significantly across the assessment points (*p* = 0.0075). The median PN concentration increased from 42.01 nmol/mg at T0 to 59.11 nmol/mg at T1 and subsequently decreased to 34.15 nmol/mg at T6. Post hoc analysis showed a significant increase between T0 and T1 and a significant reduction between T1 and T6 (both *p* < 0.05). At T12, the median PN concentration was 42.32 nmol/mg and did not differ significantly from the other time points after correction for multiple comparisons (Table 2, Figure 3).

### 3.3. Between-Group Comparison of Salivary Biomarker Concentrations

No between-group difference in AOPP concentration was observed at T0. However, AOPP levels were significantly lower in the PDT group than in the CT group at T1 (277.1 vs. 936.1 pmol/mg, *p* = 0.0004), T6 (167.7 vs. 547.6 pmol/mg, *p* = 0.0074), and T12 (275.0 vs. 478.3 pmol/mg, *p* = 0.0428) (Table 3).

Baseline AGE concentrations were comparable between the groups. At T1, the median AGE concentration was significantly higher in the PDT group than in the CT group (118.6 vs. 59.89 AFU/mg, *p* = 0.0026). No difference was detected at T6, whereas at T12, AGE concentration was significantly lower in the PDT group (42.15 vs. 69.20 AFU/mg, *p* = 0.0238).

NT concentrations did not differ between the groups at T0. At T1, a significantly lower median NT concentration was observed in the PDT group compared with the CT group (1.191 vs. 2.260 nmol/mg, *p* = 0.0212). No significant between-group differences were found at T6 or T12.

For PN, the groups differed significantly at baseline, with a higher median concentration in the PDT group than in the CT group (47.57 vs. 42.01 nmol/mg, *p* = 0.0347). Importantly, this baseline imbalance should be taken into account when interpreting subsequent between-group comparisons. No statistically significant between-group differences were observed at T1, T6, or T12.

### 3.4. Correlations Between Salivary Biomarker Concentrations, Lesion Area, and VAS Scores

Clinical outcomes for this cohort have been reported previously [22]. Briefly, median lesion area decreased in both groups immediately after treatment. In the PDT group, this reduction was maintained at T6 and T12, with a median lesion area of 0 cm^2^, whereas in the CT group it increased to 2.0 cm^2^ at both follow-up visits (Table 4).

VAS scores also declined after treatment, with lower median values at T6 and T12 in the PDT group (0.70 and 0.55, respectively) than in the CT group (2.0 at both time points) (Table 5).

In the PDT group, no statistically significant correlations were identified between salivary AOPP, AGE, NT, or PN concentrations and either lesion size or VAS scores at any assessment point (all *p* > 0.05). The strongest, although non-significant, association was observed at T6 between PN concentration and lesion size (*r* = 0.401, *p* = 0.080). All remaining correlations were weak or moderate and did not reach statistical significance (Table 6).

In the CT group, a significant positive correlation was observed at T1 between NT concentration and lesion size (*r* = 0.474, *p* = 0.041). No other significant associations were found between salivary biomarker concentrations and lesion size or VAS scores at any time point. The correlation between PN and lesion size at T6 was positive but did not reach statistical significance (*r* = 0.406, *p* = 0.084) (Table 7).

## 4. Discussion

OLP is a chronic immune-mediated disease of the oral mucosa with a recurrent course and a recognized potential for malignant transformation [33]. Because OLP is associated with broader immune and systemic disturbances, local treatment is primarily aimed at reducing the extent and severity of oral lesions and alleviating related symptoms rather than eliminating the underlying disease. Complete and permanent resolution may therefore be difficult to achieve with local therapy alone. Topical corticosteroids remain the first-line treatment because of their effectiveness in reducing lesion severity and pain [17,18]. Their prolonged or repeated use, however, may lead to oral candidiasis, mucosal atrophy, and delayed healing [19]. These limitations have contributed to the growing interest in PDT as a minimally invasive treatment with a favorable efficacy and safety profile [34].

Oxidative and nitrosative stress appear to contribute to the pathogenesis of OLP [8,10]. Previous studies have reported increased concentrations of MDA, AOPP, NO, and NO metabolites, accompanied by reduced total antioxidant capacity and disturbances in both enzymatic and non-enzymatic antioxidant defense systems [10,35,36,37]. Increased iNOS expression and the accumulation of NT and 8-nitroguanine have also been detected in the basal and suprabasal epithelial layers, corresponding to the sites of the most pronounced keratinocyte injury [36].

The present study complements our previous analyses of a partially overlapping cohort, which showed that PDT and CT affected global redox indices and selected components of the salivary antioxidant system [25,26]. In this study markers of oxidative protein modification, glycoxidation, protein nitration, and PN-related activity were examined. The observed changes were not uniform across the biomarkers, suggesting that PDT and CT influence individual components of the salivary redox environment differently.

AOPP are relatively stable products of oxidative protein modification and are considered indicators of accumulated protein damage [13]. In the PDT group, their concentrations decreased immediately after treatment and remained significantly below baseline at both follow-up visits. AOPP concentrations were also lower after PDT than after CT at T1, T6, and T12. Thus, among the biomarkers investigated, AOPP showed the clearest and most sustained response to PDT.

Although PDT is based on the local production of reactive oxygen species, the photochemical reaction is transient and restricted to the irradiated area [38]. The longer-term decrease in AOPP is therefore unlikely to reflect the immediate photochemical phase of treatment. Instead, it may result from reduced inflammatory activity, less epithelial damage, and a lower rate of persistent ROS generation after healing of the lesions. Removal of pathologically altered cells may also reduce the release of proteins susceptible to further oxidation in the oral environment.

Some support for this explanation is provided by Cosgarea et al. (2020), who demonstrated that PDT reduced the number of circulating activated CD4+CD137+ and CD8+CD137+ T lymphocytes and decreased local CD4+ and CD8+ T-cell infiltration in OLP lesions [39]. Attenuation of chronic T-cell-mediated inflammation could subsequently limit ROS production and oxidative protein modification. However, the biological significance of reduced CD137 expression should be interpreted with caution. CD137 is a costimulatory receptor involved in T-cell activation and antitumor immune responses, and increased intratumoral 4-1BB expression has been associated with enhanced lymphocytic infiltration and, in some HNSCC cohorts, improved survival [40,41]. Nevertheless, these observations originate from established malignant disease and cannot be directly extrapolated to OLP, in which CD137+ lymphocytes primarily reflect ongoing immune-mediated inflammation. Thus, the PDT-induced reduction in activated CD137+ T cells may indicate suppression of pathological inflammatory activity rather than impairment of antitumor immunity.

In contrast to PDT, CT did not produce a significant change in AOPP concentrations. This result may seem inconsistent with the anti-inflammatory action of glucocorticoids, which inhibit NF-κB signaling and reduce the expression of cytokines and other inflammatory mediators [42,43]. In our previous analysis, CT was also associated with a significant reduction in TOS [26]. Nevertheless, TOS and AOPP describe different aspects of the redox environment. TOS reflects the overall oxidizing capacity of a sample at the time of measurement, whereas AOPP represent protein modifications that have already formed and may persist until the affected proteins are cleared [44,45]. Therefore, a decrease in the current oxidant burden does not necessarily result in an immediate reduction in the concentration of previously modified proteins.

The numerical increase in AOPP after CT was not statistically significant and should not be regarded as evidence of treatment-induced oxidative damage. However, the persistent differences between the treatment groups suggest that the two interventions may differ in their longer-term effects on oxidative protein modification.

AGE followed a different pattern. In the PDT group, concentrations increased at T1 and subsequently decreased at T6 and T12. Compared with CT, AGE concentrations were higher after PDT at T1 but lower at T12. No significant variation over time was observed in the CT group.

The early rise in AGE after PDT may be related to the repeated generation of ROS during the five weekly treatment sessions. Oxidative reactions might promote the formation of reactive carbonyl and dicarbonyl compounds and thereby enhance glycoxidation [46]. Because T1 was assessed immediately after completion of the PDT protocol, the increase may represent a cumulative response to several transient episodes of photochemical ROS production.

After the final treatment session, this additional source of ROS was no longer present. AGE-modified proteins could then have been gradually removed through protein turnover and salivary clearance. The lower AGE concentration in the PDT group at T12 is consistent with a longer-lasting improvement in the local redox environment, although this interpretation cannot be established from the present data alone.

The analytical characteristics of the AGE assay must also be considered. The spectrofluorometric method measured total AGE-related fluorescence rather than individual glycation or glycoxidation products. The results may therefore have been affected by other fluorescent protein modifications.

NT is a relatively stable product of tyrosine nitration and is widely used as an indicator of nitrative stress. Although it may be produced through radicals derived from PN, peroxidase- and nitrite-dependent reactions can also contribute to its formation. NT should therefore be interpreted as a marker of protein nitration rather than as a specific measure of PN activity [12,47].

NT concentrations in the PDT group varied significantly over time in the overall analysis. Median values decreased after treatment and remained below baseline at T12, but none of the individual comparisons retained significance after correction for multiple testing. The overall result therefore indicates temporal variation without identifying a specific pair of visits responsible for the difference.

At T1, NT concentrations were lower in the PDT group than in the CT group, despite comparable baseline values. This may reflect a more rapid reduction in the conditions that favor protein nitration. By decreasing the activity of T lymphocytes and other inflammatory cells, PDT could reduce iNOS expression, NO production, and superoxide generation [39]. Lower availability of NO and superoxide would, in turn, limit the reactions leading to PN formation and tyrosine nitration.

NT concentrations did not change significantly over time in the CT group. As with AOPP, the anti-inflammatory effect of treatment may reduce ongoing nitrative reactions without immediately removing nitrated proteins already present in saliva. The between-group difference was confined to T1 and was no longer evident at T6 or T12. It therefore suggests a short-term difference between the interventions rather than a sustained effect of PDT on protein nitration.

PN showed the least consistent pattern. No significant variation was observed in the PDT group, whereas in the CT group PN increased between T0 and T1 and then decreased between T1 and T6.

Several factors complicate the interpretation of PN measurements. PN is highly reactive and has a very short lifetime in biological systems, which makes direct quantification difficult [48]. Its measurement may also be affected by the interval between collection and processing, sample temperature, storage conditions, and repeated freeze–thaw cycles. In the present study, the samples were cooled rapidly, centrifuged promptly, and stored at −80 °C, but some loss or transformation of reactive compounds before analysis cannot be excluded.

The correlation analysis showed little evidence that the investigated biomarkers were directly related to the clinical severity of OLP. In the PDT group, none of the concentrations of AOPP, AGE, NT, or PN correlated significantly with lesion area or VAS scores at any of the assessment points.

In the CT group, the only significant association was a moderate positive correlation between NT concentration and lesion area at T1. As no correction for multiple comparisons was applied to the correlation analyses, this finding should be considered exploratory and hypothesis-generating and interpreted with caution. One possible explanation is that patients with a larger residual lesion area after CT may have had a greater area of ongoing interaction between damaged epithelium and the inflammatory infiltrate. Persistent iNOS activity and RNS generation within these lesions could potentially contribute to higher levels of protein nitration.

As a stable protein modification, NT may reflect cumulative nitrative activity across a larger area of affected mucosa. In contrast, AOPP, AGE, and PN may be influenced more strongly by the overall composition of whole saliva, protein turnover, and biochemical processes occurring throughout the oral cavity. These factors may weaken their association with the dimensions of individual lesions.

Nevertheless, the correlation between NT and lesion area was detected only at T1 and was absent during follow-up. Because multiple correlations were tested, a chance finding cannot be excluded. The association should be verified in a larger cohort before any clinical relevance is attributed to it.

No significant correlations were found between the biomarkers and VAS scores. This is not unexpected, given the subjective and multifactorial nature of pain and burning in OLP. Symptom intensity depends not only on lesion size but also on lesion phenotype, particularly the presence of erythematous and erosive areas, inflammatory sensitization of mucosal nociceptors, and individual differences in pain perception [49]. Biochemical markers of accumulated molecular damage may therefore change independently of patients’ perceived symptoms.

Taken together, the correlation results suggest that salivary AOPP, AGE, NT, and PN provide information about the broader oral redox environment rather than serving as direct indicators of lesion area or symptom intensity. Clinical improvement may also occur more rapidly than the removal or turnover of oxidatively and nitratively modified proteins. However, in the absence of a healthy control group, it is difficult to determine how the observed biomarker levels relate to physiological concentrations and the extent to which they remained outside the normal range during follow-up. The isolated association between NT and lesion area after CT remains exploratory and requires confirmation in a larger independent population.

### Limitations and Future Directions

Several limitations should be considered when interpreting the present findings. First, although the sample size was determined by an a priori power calculation, the final study groups remained relatively small. This may have reduced the ability to detect modest treatment effects and may have contributed to baseline imbalances, such as the difference in PN concentrations between the groups. The limited sample size also restricts the generalizability of the results and increases the potential influence of interindividual variability.

Second, the analysis included only selected markers of oxidative and nitrative stress. Although AOPP, AGE, NT, and PN represent different aspects of molecular damage, they do not provide a comprehensive assessment of the complex redox processes involved in OLP. Additional measurements of ROS and RNS generation, antioxidant capacity, lipid and DNA oxidation, carbonyl stress, and inflammatory mediators would allow a more complete interpretation of treatment-related changes.

Third, T1 corresponded to different intervals from treatment initiation in the two groups, namely 5 weeks for PDT and 2 weeks for CT. Consequently, the differences observed at this time point may reflect not only treatment-specific effects but also differences in treatment duration and biomarker kinetics. This limits causal interpretation of the early between-group comparisons.

Potential confounding factors represent another limitation. Although factors such as smoking and the use of antioxidant supplements were controlled to some extent, redox biomarkers may also be influenced by diet, metabolic status, oral hygiene, periodontal inflammation, medication use, salivary flow, and other patient-dependent characteristics. Complete standardization of these variables is difficult, and residual confounding cannot be excluded.

The use of nonparametric statistical methods and median-based summaries was appropriate because of the non-normal distributions and wide ranges of the biomarker values. However, these approaches do not eliminate the considerable biological variability inherent in salivary biomarker research. The marked interindividual differences observed in the present cohort may therefore have obscured weaker associations and treatment effects.

Finally, the absence of a healthy control group prevented comparison of the biomarker concentrations with physiological reference values. It therefore remains unclear whether the observed post-treatment changes represented normalization toward levels found in healthy individuals or only relative changes within the OLP population.

Future studies should include larger, multicenter cohorts and an appropriately matched healthy control group. A broader panel of oxidative, nitrosative, carbonyl, and inflammatory biomarkers should be assessed using complementary and, where possible, more specific analytical methods. Standardized sampling at identical intervals from treatment initiation would improve direct comparisons between PDT and CT. Future research should also incorporate detailed assessment of potential confounders and examine whether biomarker profiles differ according to OLP phenotype, lesion severity, treatment response, and long-term clinical outcome.

## 5. Conclusions

Photodynamic therapy and topical corticosteroid treatment were associated with different patterns of change in salivary redox biomarkers in patients with oral lichen planus. Photodynamic therapy was associated with a sustained reduction in advanced oxidation protein products and with a transient increase followed by a longer-term decrease in advanced glycation end products. Lower 3-nitrotyrosine concentrations were also observed after photodynamic therapy than after corticosteroid treatment at the first post-treatment assessment; however, this assessment was performed after five weeks in the photodynamic therapy group and after two weeks in the corticosteroid group and therefore should not be interpreted as a direct time-matched comparison. The peroxynitrite findings should also be interpreted cautiously because baseline concentrations differed between the treatment groups, limiting conclusions regarding treatment-related differences.

Overall, the weak-to-modest correlations between salivary biomarkers, lesion area, and pain intensity suggest that these markers may reflect broader molecular changes rather than directly mirror clinical disease severity.

These findings should be considered exploratory and require confirmation in larger studies including broader biomarker panels and healthy control groups.

## Figures and Tables

**Table 1:** Demographic characteristics of the study participants.

Gender		
PDT	Male	2
	Female	18
CT	Male	5
	Female	14
Age		
Average (M ± SD)	PDT	60.02 ± 9.96
	CT	59.24 ± 9.12
Min-Max	PDT	29–88
	CT	34–78
Lesion type		
PDT	Reticular	17
	Erosive	3
CT	Reticular	17
	Erosive	2

**Table 2:** Time-dependent changes in salivary AOPP, AGE, NT, and PN concentrations in the PDT and CT groups. Data are presented as medians, interquartile ranges (Q1–Q3), and minimum–maximum values. *p*-values were calculated using the Friedman test.

Parameter	Measure	T0	T1	T6	T12	Friedman *p*
AOPP (pmol/mg)	Median PDT	732.9	277.1	167.7	275.0	
	Q1–Q3 PDT	470–1305	67.85–406.4	75.64–339.3	105.1–475.3	<0.0001
	Min–Max PDT	92.01–3871	31.18–2887	13.3–1012	16.12–2032	
	Median CT	828.9	936.1	547.6	478.3	
	Q1–Q3 CT	338.5–2032	73.89–2134	174.2–1654	208.5–1390	0.421770
	Min–Max CT	21.76–5023	73.89–3233	56.08–4087	36.82–5097	
AGE (AFU/mg)	Median PDT	65.38	118.6	54.55	42.15	
	Q1–Q3 PDT	52.33–87.99	76.28–187.0	36.84–98.71	29.41–60.65	<0.0001
	Min–Max PDT	30.5–192.0	21.0–520.2	22.65–213.9	19.88–432.7	
	Median CT	68.58	59.89	56.54	69.2	
	Q1–Q3 CT	42.01–90.01	45.32–77.42	44.74–96.38	36.85–129.6	0.363324
	Min–Max CT	31.79–213.9	32.2–360.2	15.23–147.2	7.806–494.2	
NT (nmol/mg)	Median PDT	2.843	1.191	1.613	1.15	
	Q1–Q3 PDT	0.99–5.085	0.59–2.076	0.761–3.805	0.411–2.323	0.046427
	Min–Max PDT	0.241–13.29	0.093–7.062	0.067–8.513	0.094–10.24	
	Median CT	2.107	2.260	2.066	1.171	
	Q1–Q3 CT	0.83–5.128	1.318–9.63	0.932–6.073	0.745–5.475	0.453811
	Min–Max CT	0.132–11.11	0.12–9.63	0.182–7.715	0.499–12.14	
PN (nmol/mg)	Median PDT	47.57	38.74	45.27	39.63	
	Q1-Q3 PDT	38.52–154.9	26.59–85.12	30.47–121.4	26.77–84.10	0.103174
	Min–Max PDT	26.78–364.1	18.16–198.7	8.357–221.1	14.91–281.3	
	Median CT	42.01	59.11	34.15	42.32	
	Q1-Q3 CT	22.84–69.82	35.59–145.9	25.63–55.25	29.2–160.2	0.007492
	Min–Max CT	10.06–179.5	20.3–207.8	13.46–228.4	17.98–276.0	

**Table 3:** Between-group comparison of salivary AOPP, AGE, NT, and PN concentrations in the PDT and CT groups at baseline (T0), immediately after treatment (T1), and at 6-month (T6) and 12-month (T12) follow-ups. Data are presented as medians. *p*-values were calculated using the Mann–Whitney U test.

Parameter	Time Point	PDT Median	CT Median	*p*-Value (Mann–Whitney)
AOPP (pmol/mg protein)	T0	732.9	828.9	0.9999
	T1	277.1	936.1	0.0004
	T6	167.7	547.6	0.0074
	T12	275.0	478.3	0.0428
AGE (AFU/mg protein)	T0	65.38	68.58	0.9060
	T1	118.6	59.89	0.0026
	T6	54.55	56.54	0.9283
	T12	42.15	69.2	0.0238
NT (nmol/mg protein)	T0	2.843	2.107	0.6465
	T1	1.191	2.26	0.0212
	T6	1.613	2.066	0.3614
	T12	1.15	1.171	0.1913
PN (nmol/mg protein)	T0	47.57	42.01	0.0347
	T1	38.74	59.11	0.2061
	T6	45.27	34.15	0.1415
	T12	39.63	42.32	0.4780

**Table 4:** Changes in lesion area in the PDT and CT groups at baseline (T0), immediately after treatment (T1), and at 6-month (T6) and 12-month (T12) follow-ups. Data are presented as mean, standard deviation (SD), median, minimum, maximum, and lower and upper quartiles.

Time Point	Group	Mean (cm^2^)	SD	Median	Minimum	Maximum	Lower Quartile	Upper Quartile
T0	PDT	3.938	2.315	3.6	0.41	9.5	2.25	4.738
T1		1.62	1.417	1.1	0.00	5.00	0.975	2.00
T6		0.7205	1.345	0.00	0.00	4.5	0.00	0.813
T12		0.6875	1.216	0.00	0.00	4.00	0.00	0.938
T0	CT	4.08	2.46	3.00	0.95	8.25	2.25	6.125
T1		1.42	1.51	1.00	0.00	4.5	0.00	2.375
T6		2.19	1.85	2.00	0.00	6.00	0.9	2.875
T12		2.20	1.81	2.00	0.00	6.00	1.00	2.75

**Table 5:** Changes in visual analog scale (VAS) scores in the PDT and CT groups at baseline (T0), immediately after treatment (T1), and at 6-month (T6) and 12-month (T12) follow-ups. Data are presented as medians, minimum–maximum values, and lower and upper quartiles.

Time Point	Group	Median	Minimum	Maximum	Lower Quartile	Upper Quartile
T0	PDT	3.55	2.00	8.00	3.00	3.00
T1		1.30	0.00	2.00	1.00	2.00
T6		0.70	0.00	3.00	0.00	1.00
T12		0.55	0.00	2.00	0.00	1.00
T0	CT	3.00	3.00	6.00	3.00	4.00
T1		2.00	0.00	4.00	0.00	2.00
T6		2.00	0.00	3.00	1.00	3.00
T12		2.00	0.00	5.00	2.00	3.00

**Table 6:** Correlations between salivary AOPP, AGE, NT, and PN concentrations and lesion area and visual analog scale (VAS) scores in the PDT group at baseline (T0), immediately after treatment (T1), and at 6-month (T6) and 12-month (T12) follow-ups. Correlations were assessed using Spearman’s rank correlation coefficient.

		Lesion Size		VAS	
Time Point	Biomarker	r	*p*	r	*p*
T0	AOPP	0.346	0.135	0.273	0.245
	AGE	0.178	0.452	0.150	0.527
	NT	−0.031	0.897	0.221	0.348
	PN	0.227	0.335	0.168	0.479
T1	AOPP	0.036	0.879	0.016	0.945
	AGE	−0.081	0.735	−0.225	0.340
	NT	0.143	0.548	0.061	0.800
	PN	0.140	0.556	−0.137	0.565
T6	AOPP	−0.125	0.599	−0.368	0.111
	AGE	0.173	0.466	0.138	0.561
	NT	0.014	0.953	0.321	0.167
	PN	0.401	0.080	0.165	0.488
T12	AOPP	0.022	0.926	−0.015	0.949
	AGE	0.027	0.910	−0.122	0.609
	NT	0.106	0.657	−0.084	0.726
	PN	0.239	0.310	−0.038	0.874

**Table 7:** Correlations between salivary AOPP, AGE, NT, and PN concentrations and lesion area and visual analog scale (VAS) scores in the CT group at baseline (T0), immediately after treatment (T1), and at 6-month (T6) and 12-month (T12) follow-ups. Correlations were assessed using Spearman’s rank correlation coefficient.

		Lesion Size		VAS	
Time Point	Biomarker	r	*p*	r	*p*
T0	AOPP	−0.097	0.691	−0.138	0.574
	AGE	0.113	0.645	0.064	0.795
	NT	0.003	0.991	0.303	0.207
	PN	0.125	0.610	−0.124	0.613
T1	AOPP	−0.038	0.879	0.009	0.972
	AGE	0.168	0.492	−0.162	0.507
	NT	0.474	0.041	0.180	0.462
	PN	−0.055	0.822	−0.091	0.712
T6	AOPP	0.216	0.375	0.002	0.993
	AGE	0.191	0.433	−0.119	0.629
	NT	0.359	0.131	−0.035	0.887
	PN	0.406	0.084	−0.174	0.477
T12	AOPP	0.140	0.569	−0.298	0.215
	AGE	−0.074	0.763	−0.002	0.994
	NT	−0.029	0.906	−0.028	0.910
	PN	−0.178	0.467	−0.178	0.295

## Data Availability

The original contributions presented in this study are included in the article. Further inquiries can be directed to the corresponding authors.
